# A systematic review assessing the existence of pneumothorax-only variants of *FLCN*. Implications for lifelong surveillance of renal tumours

**DOI:** 10.1038/s41431-021-00921-x

**Published:** 2021-07-15

**Authors:** Kenki Matsumoto, Derek Lim, Paul D. Pharoah, Eamonn R. Maher, Stefan J. Marciniak

**Affiliations:** 1 Department of Respiratory Medicine, University of Cambridge, Addenbrooke’s Hospital, Cambridge, UK; 2 Clinical Genetics Department, Birmingham Women’s and Children’s NHS Foundation Trust, Edgbaston, Birmingham UK; 3 CRUK Department of Oncology, University of Cambridge, Strangeways Research Laboratory, Cambridge, UK; 4 Department of Medical Genetics, University of Cambridge and NIHR Cambridge Biomedical Research Centre, and Cancer Research UK Cambridge Centre, Cambridge Biomedical Campus, Cambridge, UK; 5 Cambridge Institute for Medical Research (CIMR), University of Cambridge, Cambridge, UK

**Keywords:** Clinical genetics, Respiratory tract diseases

## Abstract

Individuals with Birt–Hogg–Dubé syndrome (BHDS) may develop fibrofolliculomas, pneumothorax and/or renal cell carcinoma (RCC). Currently, all patients with pathogenic *FLCN* variants are recommended to have renal surveillance. It has however been suggested that some *FLCN* variants only cause pneumothorax, which would make surveillance unnecessary in certain cases. This review assesses this possibility. We provide an up-to-date analysis of clinical and genetic features of BHDS. The PUBMED database was systematically searched to find all articles describing patients with pathogenic *FLCN* variants. The relevant clinical and genetic features of these patients were recorded and analysed. The prevalence of pneumothorax, pulmonary cysts, RCC and characteristic skin lesions in BHDS were 50.9% (*n* = 1038), 91.9% (*n* = 720), 22.5% (*n* = 929) and 47.9% (*n* = 989), respectively. There was a higher prevalence of pneumothoraces (*p* < 0.0001) but lower prevalence of dermatological findings (*p* < 0.0001) in patients from East Asia compared to North America or Europe. Of the 194 pathogenic *FLCN* variants, 76 could be defined as ‘pneumothorax-only’. Pneumothorax only pathogenic variants (POPVs) were distributed throughout the gene, and there were no statistical differences in variant type. The majority of POPVs (65/76) affected no more than three individuals. Individuals with ‘POPVs’ also tended to be younger (45 vs. 47 years, *p* < 0.05). Many apparent POPVs in the literature could result from variable expressivity, age-related penetrance and other confounding factors. We therefore recommend that all individuals found to carry a pathogenic *FLCN* variant be enroled in lifelong surveillance for RCC.

## Introduction

Pneumothorax indicates air in the pleural space. When this occurs without trauma or obvious lung pathology, the patient is said to suffer from a primary spontaneous pneumothorax. In 10% of cases, there is a family history of pneumothorax and subsequent investigation can uncover a variety of syndromic causes [1]. Birt–Hogg–Dubé syndrome (BHDS) is the most common genetic disorder diagnosed in individuals with familial pneumothorax [1]. It is caused by variants in the *FLCN* gene, which encodes the protein folliculin. *FLCN* comprises 14 exons and many pathogenic variants have been identified. Although these span the entire length of the coding sequence, several mutational hotspots exist, notably a polycytosine tract in exon 11 where insertion/deletion variants accounts for nearly 50% of all pathogenic *FLCN* variants in some cohorts [2].

*FLCN* variants are associated with the development of basal pulmonary cysts that are prone to rupture causing pneumothorax. More importantly, patients with BHDS are at increased risk of developing renal cancer (lifetime risk 25–30%). Since renal malignancy can often be cured by early surgery, annual surveillance for small malignant renal tumours has been recommended in individuals with BHDS [3]. In addition to individuals diagnosed with BHDS, pathogenic *FLCN* variants have also been described in individuals with familial pneumothorax but no other features of BHDS [4–6]. In some cases, it has been suggested that specific *FLCN* variants could result in a pneumothorax-only phenotype [4–6]. Most *FLCN* variants are truncating and will therefore cause loss of function. By contrast, variants that retain partial function, for example some missense variants, might lead to a milder phenotype. Indeed, truncating variants in the last exon and within the last 50 nucleotides of the penultimate gene have previously shown to escape nonsense-mediated decay [7].

If such genotype–phenotype correlation really exists, individuals with ‘pneumothorax-only’ pathogenic variants (POPVs) could, in theory, be spared lifelong renal surveillance, allowing medical resources to be better targeted and reducing the risk of screening-associated anxiety. To evaluate the implications of putative POPVs in *FLCN* for clinical practice, we performed a systematic review of genotype–phenotype relationships in published cases of this rare inherited disease. In particular, we investigated whether there was a difference in certain characteristics between putative POPVs and non-POPVs (variant type, location, age of patients, number of patients with that variant) to determine whether the presence of POPVs has biological plausibility or is likely an artefact.

## Methods

### Literature review

Following the Preferred Reporting Items for Systematic Reviews and Meta-Analyses (PRISMA) guidelines, we ensured that all included studies had obtained ethical approval. The PUBMED database was searched from 1st July 1974 to 1st March 2021 for English language articles using the keywords ‘Birt–-Hogg–-Dubé’ and ‘Hornstein–Knickenberg’, the latter being an alternate name for BHDS. The references list of the articles thus identified were then searched to identify additional relevant papers. Only articles reporting patients with genetically confirmed pathogenic *FLCN* variants were included. *FLCN* variants deemed not to be at least ‘likely pathogenic’ by the LOVD^3^ database or ACMG guidelines were excluded. Case reports and case series were included because they often contained novel variants that were not identified in larger cross-sectional studies. We also updated the European BHD Mutation Database (LOVD^3^) to contain all data described (both phenotypic and genotypic) [8] and papers were referenced in this database accordingly.

### Data extraction

The anonymised patient details from the 158 articles were recorded on an Excel spreadsheet. Where available the following were noted: patient’s age, sex, phenotypes (pneumothorax, cyst, skin changes, RCC), age when each phenotype was first noted, *FLCN* variant type, country where diagnosis was made and family history. This information was extracted from each paper. Not all papers reported the presence or absence of each phenotype resulting in missing data. In these situations, the particular phenotype is recorded as ‘data not available’. Available data are presented as a proportion with denominators representing individuals for whom relevant data were available. When details of individuals matched in two publications, the publications were scrutinised for evidence of potential duplication. To minimise selection bias, care was taken when recording the presence or absence of phenotypes. The presence or absence of RCC and lung cysts was recorded only if an abdominal ultrasound or CT were recorded as performed. Presence or absence of dermatological findings was recorded only if dermatological examination was explicitly noted. The speciality of the studies’ authors was noted to analyse potential bias between studies.

We wished to test for the existence of *FLCN* variants associated with pneumothorax but not renal cell carcinomas. We were less concerned to subdivide further these by skin pathology, because only renal cancer necessitates surveillance imaging. We therefore chose not to include skin phenotypes in our definition of POPV as a variant reported to cause pneumothorax but not renal carcinoma.

### Statistical analysis

Individuals were often not independent from each other as they belonged to the same family and therefore statistical models that accounted for this were used. Differences between mutually exclusive classes were examined by a mixed effects logistic regression model with a random-effects term for family. A linear mixed model with a random-effects term for family was used to compare two groups when the dependent variable was not normally distributed.

## Results

**Fig. 1:**
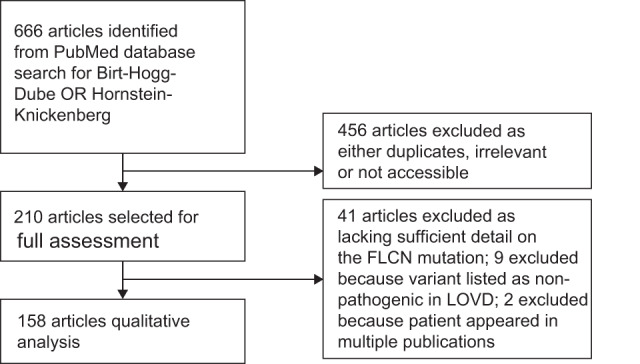
Study selection PRISMA diagram. Articles published between 1st July 1974 and 1st March 2021 were identified in PUBMED and screened as identified.

We identified 666 papers on BHDS. Of these, 158 met the inclusion criteria by reporting genetically confirmed pathogenic *FLCN* variants (Fig. 1). A total of 1059 individuals from 575 families were thus identified. Patients ranged in age from 2 to 92 years (median 46 years); 45.7% were male, and 68.8% had a confirmed family history of BHDS. Geographically, 32.7% were diagnosed in North America, 31.7% in Europe and 33.3% in E-Asia (Japan, South Korea, China and Taiwan).

### Clinical features

Overall, 50.9% (528/1038) of reported individuals had experienced at least one pneumothorax (Table 1) [2, 4–6, 8–41]. Of these, 66.7% (220/330) had recurrent pneumothoraces. There was no lateralisation of the pneumothoraces (46.4% left, 53.6% right). The median age at first pneumothorax was 34 years (range 10–78 years; *n* = 257), while the median age of individuals at the time of their case report was 46 years (range 14–92 years; *n* = 342). Of the patients who underwent thoracic CT imaging, 92.1% (662/720) had reported lung cysts. Of these patients, 63.7% had a pneumothorax [2, 4–6, 8–18, 21–28, 30, 31, 34–41].

**Table 1:** Summary of the clinical features and *FLCN* variants in patients with BHDS.

Feature	Frequency % (*n*)	Age first noted, median, range (*n*)	Age at report, median, range (*n*)	Geography % (*n*)			Genotype % (*n*)					References
				NA	EU	EA	I	MS	FS/N	LD	T	
Pulmonary cysts	91.9 (720)	N/A	46, 14–85 (394)	89.0 (245)	88.2 (152)	96.8 (312)	89.5 (86)	90.7 (43)	93.0 (571)	75.0 (12)	75 (8)	[2, 4–6, 8–18, 20–41]
Pneumothorax	50.9 (1038)	34, 10–78 (257)	46, 14–92 (344)	35.1 (342)	44.6 (336)	73.9 (337)	52.3 (109)	62.3 (53)	50.8 (834)	39.4 (34)	11.1 (9)	[2, 4–6, 8–18, 20–41]
Renal malignancy	22.5 (929)	47, 14–83 (108)	52, 14–92 (121)	23.9 (314)	27.1 (306)	16.4 (286)	29.0 (107)	25.0 (56)	21.7 (725)	18.8 (32)	11.1 (9)	[2, 6, 8, 10–18, 20, 21, 24–29, 31–41]
Dermatological manifestations	47.9 (989)	38, 20–65 (44)	51, 22–92 (213)	73.4 (342)	50.3 (304)	20.6 (320)	65.4 (104)	26.9 (52)	47.5 (794)	26.7 (30)	77.8 (9)	[2, 4–6, 8–18, 20–41]

*NA* North America, *EU* Europe, *EA* East Asia, *I* intronic, *MS* missense/in-frame deletion, *FS/N* frameshift/nonsense, *LD* large deletion/duplication, *T* transcription initiation variant.

Of the 989 patients with recorded dermatological examinations, 47.9% had lesions consistent with BHDS [2, 4–6, 8–18, 20–41]. Most had fibrofolliculomas (90.5%; 429/474), 8.8% (42/474) had trichodiscomas and 4.0% (19/474) had perifollicular adenomas. The median age at which skin changes were first noted was 38 years (range 20–65 years; *n* = 44), while the median age of these individuals at the time of their case report was 51 years (range 22–92 years; *n* = 213).

Of the 929 patients who underwent imaging of the abdomen, 22.5% had malignant renal tumours [2, 6, 8–18, 20–29, 31–41]. In 52.5% (62/118), the tumour was unifocal and did not recur after excision. A variety of histological subtypes were reported: chromophobe 32.8% (63/192), hybrid oncocytoma-chromophobe 24.5% (47/192), clear cell 11.5% (22/192), oncocytomas 9.9% (19/192), hybrid clear cell-chromophobe 7.3% (14/192), papillary 5.2% (10/192) and the remaining patients had either a hybrid oncocytoma-clear cell or a hybrid papillary-clear cell tumour. The median age at first diagnosis of renal cell carcinoma was 47 years (range 14–83 years; *n* = 108). Their median age at the time of case report was 52 years (14–92 years; *n* = 121).

Differences were apparent in the phenotypes reported in geographical regions. The frequency of pulmonary cysts in East Asia was significantly higher (96.8%; 302/312) than North America (89.0%; 218/245) or Europe (88.2%; 134/152) (*p* < 0.0001) [2, 4–6, 8–18, 20–30, 34–41]. Similarly, a higher proportion of pneumothorax was seen in East Asian reports (73.9%; 249/337) compared with North America (35.1%, 120/342) or Europe (44.6%, 150/336) (*p* < 0.0001). By contrast, a slightly higher proportion of renal cancer was observed in European reports (27.1%, 83/306) than in either North American (23.9%, 75/314) or East Asian individuals (16.4%, 47/286) (*p* < 0.05). More strikingly, the proportion of dermatological features was far lower in East Asian (20.6%, 66/320) than in European (50.3%, 153/304) or North American cases (73.4%, 251/342) (*p* < 0.0001).

Of the individuals where the data were available, the commonest presentation was a positive family history of the disorder (44.0% of individuals, 438/995) [2, 4–6, 8–18, 20–29, 31–41]. Skin findings were the presenting feature in 17.8% (177/995) of diagnoses, followed by pneumothorax in 19.6% (195/995) and renal cancer in 7.5% (75/995) of cases. The remainder could not be linked to a single feature or were the result of incidental detection of pulmonary cysts after CT scanning. Of note, a longer latency to diagnosis was observed when pneumothorax was the first clinical feature (median time to diagnosis 6 years; *n* = 138) compared with 0 years for both RCC (*n* = 13) and skin changes (*n* = 36).

### Genetic features

**Fig. 2:**
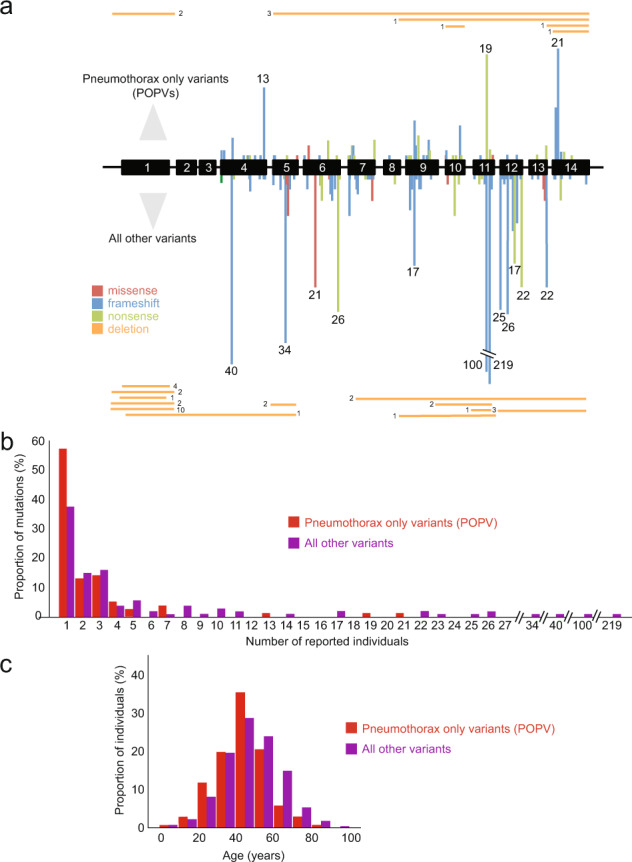
Pathogenic *FLCN* variants. **A** Missense/in-frame variants in red, frameshifts in blue, nonsense variants in light green, large deletions/duplications in orange. Bars are proportional to numbers of affected individuals, for mutational hotspots the number of individuals is given above each bar. ‘Pneumothorax-only’ pathogenic variants (POPV) shown above exons, all other variants shown below. **B** Histogram of individuals affected by POPV (red), all other variants (purple). **C** Ages of individuals reported to carry POPV (red) or all other FLCN variants (purple) (colour figure online).

A total of 194 pathogenic variants were identified from the literature [2, 4–6, 8–18, 20–42]. These comprised 132 nonsense and frameshift variants, 31 intronic variants, 11 missense variants/in-frame deletions, 13 large deletions/duplications and 7 variants affecting the initiation of transcription (Fig. 2A and Supplementary Table 1). Eleven variants had no associated patient details and so were excluded from subsequent analyses.

It has been suggested that certain *FLCN* variants lead to a forme fruste of BHDS with pneumothoraces but no renal cancers [4, 5]. Since the existence of ‘pneumothorax-only’ *FLCN* variants would have important consequences for screening protocols, we examined these in more detail. Of the 183 variants, there were 76 ‘pneumothorax-only’ variants, 24 also had skin changes (Fig. 2A).

POPVs were distributed throughout the gene (Fig. 2A) and no association was found with variant type (p = 0.78). We hypothesised that at least some *FLCN* variants might erroneously appear as POPVs if detected in younger or smaller families with less chance of having manifested renal carcinomas and were not adequately followed-up (given that the median age at first pneumothorax was 34 years and median age at RCC was 47 years). Indeed, we observed that a majority of putative POPVs (85.5%, 65/76) affected no more that three individuals (Fig. 2B, C). Furthermore, the median age of individuals with POPVs was significantly lower than those with non-pneumothorax-only variants (45 vs. 47 years, *p* < 0.01). Importantly, there were less data on elderly members (70 years or over) in families with reportedly ‘pneumothorax-only’ variants (Fig. 2C).

Of 991 individuals studied in this review, 10 were diagnosed before the age of 18 (range: 2 to 17) and a further 7 were diagnosed between the ages of 18 and 20 [8, 12, 16, 18, 33–40]. Of the ten patients diagnosed before the age of 18, six were asymptomatic (five diagnosed through family screening and one through a mutation analysis following a diagnosis of leiomyosarcoma), three presented with pneumothoraces and one presented with renal cell carcinoma. Overall, there were 14 patients with a *FLCN* variant, who had their first pneumothorax before the age of 20 [8, 12, 13, 18, 31, 32, 34–36, 41]. There was one case of renal cell carcinoma [33] and no cases of pathognomonic skin lesions. The youngest ages of presentation for pneumothorax and renal cell carcinoma were 10 and 14, respectively [33, 36].

## Discussion

From the literature, we identified 1059 individuals with pathogenic *FLCN* variants across 575 families. Although it is known that such *FLCN* variants increase the risk of pneumothorax by up to 50-fold [43], the true proportion of pneumothoraces in BHDS remains unclear. We found that 50.9% of reported individuals with *FLCN* variants had suffered at least one pneumothorax, which is considerably higher than the 30% typically quoted [43]. This difference may reflect reporting bias in the literature compared to large cross-sectional studies. It is noteworthy that many individuals (44.0%) were identified through family tracing and so many were young and yet to develop complications.

A variety of genetic disorders can present to the respiratory physician as pneumothorax. The diagnoses that can subsequently be made can lead to life-extending treatments, not for the pneumothorax but for other features of the genetic disorder. In the case of BHDS, although patients often present with pneumothorax, they are also at risk for developing potentially fatal renal cancer years later. Annual screening is currently recommended [3], but several reports of POPVs have brought into question the necessity for screening all patients with *FLCN* variants [4, 5].

As well as missense mutations, we looked at truncating variants in the last exon and the last 50 nucleotides of the penultimate exon, in particular, as they have previously shown to escape nonsense-mediated decay [7]. Such an event would result in a carboxyterminally truncated folliculin protein that might retain partial function and so generate an attenuated phenotype compared with other variants that produce little or no protein. Indeed, Park et al. [30] reported a novel *FLCN* c.1489_1490delTG pathogenic variant that escaped nonsense-mediated mRNA decay. The variant was within the last 50 base pairs of exon 13 and the proband had presented with recurrent primary spontaneous pneumothorax but no renal cell carcinoma. This observation would be consistent with the hypothesis that exon 14 variants might have a milder phenotype. In this study, however, we found no association between variant type (missense or truncating mutations in the last exon/last 50 nucleotides of exon 13) and POPVs.

Overall, although ‘apparent POPVs’ could represent true ‘pneumothorax-only variants’, it seems more likely that they are artefacts for the following reasons: (i) on average each apparent POPV is carried by fewer individuals than non-POPV variants, (ii) no unequivocal clustering of such variants was apparent in the *FLCN* gene or folliculin protein domains and (iii) POPVs were more likely to be found in younger patients who have a lower probability of manifesting age-related renal tumours. We therefore recommend that all patients with BHDS and or pathogenic variants in the *FLCN* gene are offered renal surveillance.

Our review supports reports that pulmonary manifestations of BHDS appear more frequently in East Asian populations [13]. Conversely, renal cell cancer and dermatological features seem less common in East Asians. It is unclear if these findings reflect differences in genetic or environmental factors, or differences in diagnostic pathways. It is plausible that unidentified environmental factors might play a role. For example, sun exposure is thought to predispose individuals with tuberous sclerosis to angiofibromata [44]. It is not clear, however, if similar factors affect the development of fibrofolliculomas. On the other hand, most skin examinations reported in the East Asian papers were performed by respiratory physicians, while it is known that dermatologists are threefold (23.3% vs. 73.9%) more likely to identify skin lesions in patients with BHDS [31]. Moreover, there are fewer follicular units in individuals from East Asia compared to Caucasians [45], which might lead to fewer fibrofolliculomas being present and so more likely to be overlooked.

Pneumothoraces were recurrent in 66.7% of individuals with a *FLCN* variant who previously presented with a pneumothorax. This is higher than the 30% reported for large series of spontaneous pneumothoraces [46]. It remains possible that thorough investigation of pneumothoraces and consequent diagnosis of BHDS were more likely in patients with recurrent pneumothoraces. Prospective long-term follow up of individuals identified by family tracing is necessary to determine the true recurrence rate. Renal cell carcinoma, the most sinister pathology in BHDS, had a prevalence of 22.5% consistent with previous estimates of 12–34% [32]. In contrast to pneumothorax, which had a median age of diagnosis of 34 years, renal cancer was a later complication with a median age at diagnosis of 47 years, although the range was large.

Skin changes consistent with BHDS were found in 47.9% of patients, which is considerably lower than the 85% suggested in previous reports [43]. This might reflect the increasing number of East Asian case reports, which report much lower proportions of positive skin findings. The median age at which patients first noted their skin change was 38, which is lower than previous reports [31].

The age at which predictive genetic testing in adult-onset disorders should commence has been widely discussed. It is recommended that individuals should only be tested when diagnosis would influence their overall management [47]. In the case of BHDS, genetic testing should therefore be influenced by the age of commencement of renal screening. Current guidance for BHDS recommends that renal screening begins at 20 years of age [3]. A majority of symptomatic adolescents with BHDS present with pneumothoraces rather than renal cell carcinoma, although we note that there is a report of an individual younger than 20 developing renal cancer in this disorder [33].

Previous estimates suggest that 5–10% of patients with primary pneumothoraces have BHDS, yet we observe a mean latency of 6 years between pneumothorax and diagnosis suggesting insufficient awareness amongst clinicians that will delay instigation of renal cancer screening. We previously suggested a diagnostic pathway for patients with pneumothorax by which such a delay could be avoided [1].

This systematic review suffers from a number of limitations. First, all the patient information came from cross-sectional studies. This can introduce ascertainment bias, although family tracing would still identify asymptomatic or elderly affected relatives. Further, due to the lack of patient follow-up, renal cancer, which tends to present later in life, may be underrepresented and may contribute to reports of POPVs. This is a limitation inherent in cross-sectional studies. There has yet to be a prospective analysis of large BHDS cohorts; this would improve confidence in the frequencies of phenotypes that develop with age. Second, bias might have been introduced by the clinical speciality of reporting authors. We noted that 24.4% of patients were reported by pulmonologists, 19.0% by dermatologists, 21.8% by nephrologists, 14.8% from genetic departments and the remaining 20% from a variety of departments including oncology, pathology and general medicine. However, this variety may act to minimise bias from a clinician’s specialty. Finally, there was variability in the clinical data available and the manner by which it had been collected. For example, only some examinations of the skin were performed by dermatologists, while CT examinations were available only for a subset of patients. Nevertheless, systematic analysis of all published cases is a tractable approach to the analysis of rare conditions such as BHDS.

In conclusion, this systematic review provides a comprehensive analysis of the clinical and genetic features of BHDS and is relevant to respiratory physicians to whom new patients may present with familial, sporadic and or recurrent pneumothorax. We recommend that all individuals found to carry a pathogenic *FLCN* variant be offered lifelong surveillance for renal cancer, since pneumothorax-only *FLCN* variants are likely to be rare.

## Supplementary information

Supplementary Table 1

## References

[1] Scott RM, Henske EP, Raby B, Boone PM, Rusk RA, Marciniak SJ (2018). Familial pneumothorax: towards precision medicine. Thorax.

[2] Toro JR, Wei MH, Glenn GM, Weinreich M, Toure O, Vocke C (2008). BHD mutations, clinical and molecular genetic investigations of Birt–Hogg–Dube syndrome: a new series of 50 families and a review of published reports. J Med Genet.

[3] Menko FH, van Steensel MA, Giraud S, Friis-Hansen L, Richard S, Ungari S (2009). Birt–Hogg–Dubé syndrome: diagnosis and management. Lancet Oncol.

[4] Graham RB, Nolasco M, Peterlin B, Garcia CK (2005). Nonsense mutations in folliculin presenting as isolated familial spontaneous pneumothorax in adults. Am J Respir Crit Care Med.

[5] Painter JN, Tapanainen H, Somer M, Tukiainen P, Aittomäki K (2005). A 4-bp deletion in the Birt–Hogg–Dubé gene (FLCN) causes dominantly inherited spontaneous pneumothorax. Am J Hum Genet.

[6] Kumar K, Ross C (2019). Birt–Hogg–Dubé syndrome presenting with spontaneous pneumothorax and extensive pulmonary cysts in the absence of skin lesions or renal pathology. BMJ Case Rep.

[7] White J, Mazzeu JF, Hoischen A, Jhangiani SN, Gambin T, Alcino MC (2015). DVL1 frameshift mutations clustering in the penultimate exon cause autosomal-dominant Robinow syndrome. Am J Hum Genet.

[8] LOVD3—Leiden Open Variation Database. 2020. Available from: http://www.LOVD.nl/FLCN.

[9] Furuya M, Hong SB, Tanaka R, Kuroda N, Nagashima Y, Nagahama K (2015). Distinctive expression patterns of glycoprotein non-metastatic B and folliculin in renal tumors in patients with Birt–Hogg–Dubé syndrome. Cancer Sci.

[10] Xing H, Liu Y, Jiang G, Li X, Hou Y, Yang F (2017). Clinical and genetic study of a large Chinese family presented with familial spontaneous pneumothorax. J Thorac Dis.

[11] Liu Y, Xu Z, Feng R, Zhan Y, Wang J, Li G (2017). Clinical and genetic characteristics of chinese patients with Birt–Hogg–Dubé syndrome. Orphanet J Rare Dis.

[12] Liu Y, Xing H, Huang Y, Meng S, Wang J (2019). Familial spontaneous pneumothorax: importance of screening for Birt–Hogg–Dubé syndrome. Eur J Cardiothorac Surg.

[13] Kunogi M, Kurihara M, Ikegami TS, Kobayashi T, Shindo N, Kumasaka T (2010). Clinical and genetic spectrum of Birt–Hogg–Dube syndrome patients in whom pneumothorax and/or multiple lung cysts are the presenting feature. J Med Genet.

[14] Leter EM, Koopmans AK, Gille JJ, van Os TA, Vittoz GG, David EF (2008). Birt–Hogg–Dubé syndrome: clinical and genetic studies of 20 families. J Invest Dermatol.

[15] Ren HZ, Zhu CC, Yang C, Chen SL, Xie J, Hou YY (2008). Mutation analysis of the FLCN gene in Chinese patients with sporadic and familial isolated primary spontaneous pneumothorax. Clin Genet.

[16] Kluger N, Giraud S, Coupier I, Avril MF, Dereure O, Guillot B (2010). Birt–Hogg–-Dubé syndrome: clinical and genetic studies of 10 French families. Br J Dermatol.

[17] Kluijt I, de Jong D, Teertstra HJ, Axwijk PH, Gille JJ, Bell K (2009). Early onset of renal cancer in a family with Birt–Hogg–Dubé syndrome. Clin Genet.

[18] Maffé A, Toschi B, Circo G, Giachino D, Giglio S, Rizzo A (2011). Constitutional FLCN mutations in patients with suspected Birt–Hogg–Dubé syndrome ascertained for non-cutaneous manifestations. Clin Genet.

[19] Skolnik K, Tsai WH, Dornan K, Perrier R, Burrowes PW, Davidson WJ (2016). Birt–Hogg–Dubé syndrome: a large single family cohort. Respir Res.

[20] Khoo SK, Giraud S, Kahnoski K, Chen J, Motorna O, Nickolov R (2002). Clinical and genetic studies of Birt–Hogg–Dubé syndrome. J Med Genet.

[21] Benusiglio PR, Giraud S, Deveaux S, Méjean A, Correas JM, Joly D (2014). Renal cell tumour characteristics in patients with the Birt–Hogg–Dubé cancer susceptibility syndrome: a retrospective, multicentre study. Orphanet J Rare Dis.

[22] Furuya M, Yao M, Tanaka R, Nagashima Y, Kuroda N, Hasumi H (2016). Genetic, epidemiologic and clinicopathologic studies of Japanese Asian patients with Birt–Hogg–Dubé syndrome. Clin Genet.

[23] Furuya M, Tanaka R, Koga S, Yatabe Y, Gotoda H, Takagi S (2012). Pulmonary cysts of Birt–Hogg–Dubé syndrome: a clinicopathologic and immunohistochemical study of 9 families. Am J Surg Pathol.

[24] Hoshika Y, Takahashi F, Togo S, Hashimoto M, Nara T, Kobayashi T (2016). Haploinsufficiency of the folliculin gene leads to impaired functions of lung fibroblasts in patients with Birt–Hogg–Dubé syndrome. Physiol Rep.

[25] Torricelli E, Occhipinti M, Cavigli E, Tancredi G, Rosi E, Rossi C (2019). The relevance of family history taking in the detection and management of Birt–Hogg–Dubé syndrome. Respiration.

[26] Genc Yavuz B, Guzel Tanoglu E, Salman Yılmaz S, Colak S (2019). A novel FLCN mutation in family members diagnosed with primary spontaneous pneumothorax. Mol Genet Genom Med.

[27] Benhammou JN, Vocke CD, Santani A, Schmidt LS, Baba M, Seyama K (2011). Identification of intragenic deletions and duplication in the FLCN gene in Birt–Hogg–Dubé syndrome. Genes Chromosomes Cancer.

[28] Babaei Jandaghi A, Daliri S, Kikkawa M, Khaledi M, Soleimanifar N, Alizadeh A (2013). The discovery of a Persian family with a form of Birt–Hogg–Dubé syndrome lacking the typical cutaneous stigmata of the syndrome. Clin Imaging.

[29] Schmidt LS, Nickerson ML, Warren MB, Glenn GM, Toro JR, Merino MJ (2005). Germline BHD-mutation spectrum and phenotype analysis of a large cohort of families with Birt–Hogg–Dubé syndrome. Am J Hum Genet.

[30] Park YJ, Lee SK, Kang SH, Jang SJ, Moon DS, Park G (2016). A novel FLCN c.1489_1490delTG mutation that escapes the nonsense-mediated decay system. Ann Clin Lab Sci.

[31] Iwabuchi C, Ebana H, Ishiko A, Negishi A, Mizobuchi T, Kumasaka T (2018). Skin lesions of Birt–Hogg–Dubé syndrome: clinical and histopathological findings in 31 Japanese patients who presented with pneumothorax and/or multiple lung cysts. J Dermatol Sci.

[32] Houweling AC, Gijezen LM, Jonker MA, van Doorn MB, Oldenburg RA, van Spaendonck-Zwarts KY (2011). Renal cancer and pneumothorax risk in Birt–Hogg–Dube syndrome; an analysis of 115 FLCN mutation carriers from 35 BHD families. Br J Cancer.

[33] Schneider M, Dinkelborg K, Xiao X, Chan-Smutko G, Hruska K, Huang D (2018). Early onset renal cell carcinoma in an adolescent girl with germline FLCN exon 5 deletion. Fam Cancer.

[34] Johannesma PC, van den Borne BE, Gille JJ, Nagelkerke AF, van Waesberghe JT, Paul MA (2014). Spontaneous pneumothorax as indicator for Birt–Hogg–Dubé syndrome in paediatric patients. BMC Pediatr.

[35] Ardolino L, Silverstone E, Varjavandi V, Yates D (2020). Birt–Hogg–Dubé syndrome presenting with macroscopic pulmonary cyst formation in a 15-year-old. Respirol Case Rep.

[36] Demir M, Çobanoğlu N (2016). An 18-year-old man with recurrent pneumothorax since he was 10-year-old. Pediatr Pulmonol.

[37] Bird LM, Kuo DJ, Masser-Frye D, Mo JQ, Elster JD (2020). Leiomyosarcoma in Birt–Hogg–Dubé syndrome. J Pediatr Hematol Oncol.

[38] Sprague J, Landau JW (2016). Birt–Hogg–Dubé syndrome presenting as a nevus comedonicus-like lesion in an 8-year-old boy. Pediatr Dermatol.

[39] Ray A, Paul S, Chattopadhyay E, Kundu S, Roy B (2015). Genetic analysis of familial spontaneous pneumothorax in an Indian family. Lung.

[40] Enomoto Y, Namba Y, Hoshika Y, Komemushi Y, Mitani K, Kume H (2020). A case of Birt–Hogg–Dubé syndrome implying reduced or no wild-type folliculin without mutated protein is pathogenic. Eur J Med Genet.

[41] Gunji Y, Akiyoshi T, Sato T, Kurihara M, Tominaga S, Takahashi K (2007). Mutations of the Birt–Hogg–Dube gene in patients with multiple lung cysts and recurrent pneumothorax. J Med Genet.

[42] Lim DH, Rehal PK, Nahorski MS, Macdonald F, Claessens T, Van Geel M (2010). A new locus-specific database (LSDB) for mutations in the folliculin (FLCN) gene. Hum Mutat.

[43] Schmidt LS, Linehan WM (2015). Molecular genetics and clinical features of Birt–Hogg–Dubé syndrome. Nat Rev Urol.

[44] Tyburczy ME, Wang JA, Li S, Thangapazham R, Chekaluk Y, Moss J (2014). Sun exposure causes somatic second-hit mutations and angiofibroma development in tuberous sclerosis complex. Hum Mol Genet.

[45] Tsai RY, Lee SH, Chan HL (2002). The distribution of follicular units in the Chinese scalp: implications for reconstruction of natural-appearing hairlines in Orientals. Dermatol Surg.

[46] Hallifax RJ, Goldacre R, Landray MJ, Rahman NM, Goldacre MJ (2018). Trends in the incidence and recurrence of inpatient-treated spontaneous pneumothorax, 1968–2016. JAMA.

[47] Borry P, Evers-Kiebooms G, Cornel MC, Clarke A, Dierickx K, (ESHG) PaPPCPotESoHG. (2009). Genetic testing in asymptomatic minors: background considerations towards ESHG Recommendations. Eur J Hum Genet.

