# Supplementary material for: A systematic review assessing the existence of pneumothorax-only variants of FLCN. Implications for lifelong surveillance of renal tumours
Source: Eur J Hum Genet. 2021 Jul 15;29(11):1595–600. doi: 10.1038/s41431-021-00921-x (PMC8560836; doi:10.1038/s41431-021-00921-x)
Supplement: Supplementary file 1 — Supplementary Table 1 [file 41431_2021_921_MOESM1_ESM.docx]

**Supplementary Table 1:** *FLCN* mutations with American College of Medical Genetics (ACMG) classification

| **DNA change (cDNA)** | **Overall effect on RNA*** | **Pathogenicity (ACMG)** | **ACMG Class** | **LOVD3** |
| --- | --- | --- | --- | --- |
| c.(?_-504)_(-228+1_-227-1)del | Large deletion | PVS1, PM2, PP3, PP4 | Pathogenic | Pathogenic |
| Deletion including exon 1 | Large deletion | PVS1, PS3, PM1, PM2, PP3, PP4 | Pathogenic | Pathogenic |
| c.-6544_-228+454delins-3779_-3655inv | Affecting initiation | PVS1, PM2, PP3 | Pathogenic | Pathogenic |
| c.-5575_-228+341delinsCCCCCATGG | Affecting initiation | PVS1, PM2, PP3 | Pathogenic | Pathogenic |
| c.-4174_-227-1566del | Affecting initiation | PVS1, PM2, PP3 | Pathogenic | Pathogenic |
| c.-227-853_397-295del | Affecting initiation | PVS1, PM2, PP3 | Pathogenic | Pathogenic |
| c.1A>G | Affecting initiation | PVS1, PS3, PM1, PM2 | Pathogenic | Pathogenic |
| c.3delG | Affecting initiation | PVS1, PM1, PM2, PP3 | Pathogenic | Pathogenic |
| c.3G>A | Affecting initiation | PVS1, PM1, PM2 | Pathogenic | Likely pathogenic |
| c.17_21delCTCTC | Frameshift | PVS1, PM2, PP3 | Pathogenic | Pathogenic |
| c.57_58delCT | Frameshift | PVS1, PM2, PP3, PP4 | Pathogenic | Pathogenic |
| c.59delT | Frameshift | PVS1, PM2, PP1, PP3, PP4 | Pathogenic | Pathogenic |
| c.67G > T | Nonsense | PVS1, PM2, PP3, PP4 | Pathogenic | Pathogenic |
| c.119delG | Frameshift | PVS1, PS3, PM2, PP3 | Pathogenic | Pathogenic |
| c.145G > T | Nonsense | PVS1, PM2, PP3, PP4 | Pathogenic | Pathogenic |
| c.147delA | Frameshift | PVS1, PM2, PP3, PP4 | Pathogenic | Pathogenic |
| c.155delC | Frameshift | PVS1, PM2, PM6, PP4 | Pathogenic | Pathogenic |
| c.157C > T | Nonsense | PVS1, PM2, PP3, PP4 | Pathogenic | Pathogenic |
| c.158delA | Frameshift | PVS1, PM2, PP3, PP4 | Pathogenic | Pathogenic |
| c.185delG | Frameshift | PVS1, PM2, PP3, PP4 | Pathogenic | Pathogenic |
| c.199dupG | Frameshift | PVS1, PM2, PP3, PP4 | Pathogenic | Pathogenic |
| c.214delA | Frameshift | PVS1, PM2, PP3, PP4 | Pathogenic | Pathogenic |
| c.233delA | Frameshift | PVS1, PM2, PP3, PP4 | Pathogenic | Pathogenic |
| c.235_238del | Frameshift | PVS1, PM2, PP1, PP4 | Pathogenic | Pathogenic |
| c.240delC | Frameshift | PVS1, PM2, PP3, PP4 | Pathogenic | Pathogenic |
| c.241del | Frameshift | PVS1, PM2, PP3, PP4 | Pathogenic | Pathogenic |
| c.249 + 1G > T | Intronic/splice site | PVS1, PM2, PP3, PP4 | Pathogenic | Pathogenic |
| c.249+1G>A | Intronic/splice site | PVS1, PS3, PM1, PP3 | Pathogenic | Pathogenic |
| c.(249+1_250-1)_(*1_?)del | Large deletion | PVS1, PM2, PP3, PP4 | Pathogenic | Pathogenic |
| c.250-2A>G | Intronic/splice site | PVS1, PM2, PP4 | Pathogenic | Pathogenic |
| c.250-1G>A | Intronic/splice site | PVS1, PM2, PP1, PP4 | Pathogenic | Pathogenic |
| c.250delG | Frameshift | PVS1, PM2, PP3 | Pathogenic | Pathogenic |
| c.252delC | Frameshift | PVS1, PM2, PP1, PP4 | Pathogenic | Pathogenic |
| c.252insG | Frameshift | PVS1, PM2, PP3, PP4 | Pathogenic | Pathogenic |
| c.256_269del14 | Frameshift | PVS1, PM2, PP3, PP4 | Pathogenic | Pathogenic |
| c.260C>G | Nonsense | PVS1, PM2, PP3, PP4 | Pathogenic | Pathogenic |
| c.296delA | Frameshift | PVS1, PM2, PP1, PP4 | Pathogenic | Pathogenic |
| c.318C>G | Nonsense | PVS1, PM2, PP3, PP4 | Pathogenic | Pathogenic |
| c.319_320delGTinsCAC | Frameshift | PVS1, PM2, PP4 | Pathogenic | Pathogenic |
| c.(249+1_250-1)_(396+1_397-1)del | Large deletion | PVS1, PM2, PP3, PP4 | Pathogenic | Pathogenic |
| c.323G>T | Missense/in-frame deletion | PM2, PM6, PP3, PP4, PP5 | Likely pathogenic | Likely pathogenic |
| c.328C>T | Nonsense | PVS1, PM2, PP3, PP4 | Pathogenic | Pathogenic |
| c.332_349del | Missense/in-frame deletion | PM2, PM4, PP1, PP4 | Likely pathogenic | Likely pathogenic |
| c.347dupA | Frameshift | PVS1, PM2, PP4 | Pathogenic | Pathogenic |
| c.365_372del | Frameshift | PVS1, PM2, PP3, PP4 | Pathogenic | Pathogenic |
| c.376delG | Frameshift | PVS1, PM2, PP3, PP4 | Pathogenic | Pathogenic |
| c.394G>A | Missense/in-frame deletion | PM2, PM6, PP3, PP4 | Likely pathogenic | Pathogenic |
| c.397-10_397-1del | Intronic/splice site | PVS1, PM2, PP1, PP4 | Pathogenic | Pathogenic |
| c.397-7_399del | Intronic/splice site | PVS1, PS3, PM2, PP4 | Pathogenic | Pathogenic |
| c.397-1G>C | Intronic/splice site | PVS1, PM2, PP4 | Pathogenic | PAthogenic |
| c.404delC | Frameshift | PVS1, PM2, PP3, PP4 | Pathogenic | Pathogenic |
| c.420delC | Frameshift | PVS1, PM2, PP4 | Pathogenic | Pathogenic |
| c.427_429delTTC | Missense/in-frame deletion | PS3, PM2, PM6, PP3, PP4 | Pathogenic | Pathogenic |
| c.443_459del | Frameshift | PVS1, PM2, PP3, PP4 | Pathogenic | Pathogenic |
| c.469_471delTTC | Missense/in-frame deletion | PS3, PM4, PP1, PP3 | Likely pathogenic | Likely pathogenic |
| c.494delG | Frameshift | PVS1, PM2, PP3, PP4 | Pathogenic | Pathogenic |
| c.499C>T | Nonsense | PVS1, PM2, PP3, PP4 | Pathogenic | Pathogenic |
| c.499_500 insCTGTGGATGT | Frameshift | PVS1, PM2, PP3, PP4 | Pathogenic | Pathogenic |
| c.510C>G | Nonsense | PVS1, PM2, PP3, PP4 | Pathogenic | Pathogenic |
| c.543C>G | Nonsense | PVS1, PM2, PP3, PP4 | Pathogenic | Pathogenic |
| c.563delT | Frameshift | PVS1, PM2, PP3, PP4 | Pathogenic | Pathogenic |
| c.566_577delTGCTGGGGAAGGinsCC | Frameshift | PVS1, PM2, PP3, PP4 | Pathogenic | Pathogenic |
| c.573_574delinsT | Frameshift | PVS1, PM2, PP3, PP4 | Pathogenic | Pathogenic |
| c.583G>T | Nonsense | PVS1, PM2, PP3, PP4 | Pathogenic | Pathogenic |
| c.584delG | Frameshift | PVS1, PM2, PP3, PP4 | Pathogenic | Pathogenic |
| c.601C>T | Nonsense | PVS1, PM2, PP3, PP4 | Pathogenic | Pathogenic |
| c.610_611delGCinsTA | Nonsense | PVS1, PM2, PP3, PP4 | Pathogenic | Pathogenic |
| c.616A>T | Nonsense | PVS1, PM2, PP3, PP4 | Pathogenic | Pathogenic |
| c.(618+1_619-1)_(*1_?)del | Large deletion | PVS1, PM2, PP3 | Pathogenic | Pathogenic |
| c.618+2T>A | Intronic/splice site | PVS1, PM2, PP4 | Pathogenic | Pathogenic |
| c.619-1G>A | Intronic/splice site | PVS1, PM2, PP4 | Pathogenic | Pathogenic |
| c.625G>T | Nonsense | PVS1, PM2, PP3, PP4 | Pathogenic | Pathogenic |
| c.632_633delAGinsC | Frameshift | PVS1, PM2, PP1, PP4 | Pathogenic | Pathogenic |
| c.636delC | Frameshift | PVS1, PM2, PP3, PP4 | Pathogenic | Pathogenic |
| c.639delT | Frameshift | PVS1, PM2, PP1, PP4 | Pathogenic | Pathogenic |
| c.649C>T | Nonsense | PVS1, PM2, PP3, PP4 | Pathogenic | Pathogenic |
| c.655dupG | Frameshift | PVS1, PM2, PP4 | Pathogenic | Pathogenic |
| c.658C>T | Nonsense | PVS1, PM2, PP3, PP4 | Pathogenic | Pathogenic |
| c.663dup | Frameshift | PVS1, PM2, PP4 | Pathogenic | Pathogenic |
| c.668delA | Frameshift | PVS1, PM2, PP4 | Pathogenic | Pathogenic |
| c.671_672delCA | Frameshift | PVS1, PM2, PP1, PP4 | Pathogenic | Pathogenic |
| c.747_756insGTGATGACAA | Frameshift | PVS1, PM2, PP4 | Pathogenic | Pathogenic |
| c.755dup | Frameshift | PVS1, PM2, PP3 | Pathogenic | Pathogenic |
| c.770_772del | Missense/in-frame deletion | PS3, PM2, PM4, PP4 | Likely pathogenic | Likely pathogenic |
| c.771del | Frameshift | PVS1, PM2, PP3, PP4 | Pathogenic | Pathogenic |
| c.779G>A | Nonsense | PVS1, PS3, M2, PP3, PP4 | Pathogenic | Pathogenic |
| c.779+1G>T | Intronic/splice site | PVS1, PM2, PP4 | Pathogenic | Pathogenic |
| c.780-2A>G | Intronic/splice site | PVS1, PM2, PP4 | Pathogenic | Pathogenic |
| c.780-1G>T | Intronic/splice site | PVS1, PM2, PP4 | Pathogenic | Pathogenic |
| c.823_824delGA | Frameshift | PVS1, PM2, PP3, PP4 | Pathogenic | Pathogenic |
| c.836_839del | Frameshift | PVS1, PM2, PP3, PP4 | Pathogenic | Pathogenic |
| c.862-?_1062+?del | Large deletion | PVS1, PM2, PP3, PP4 | Pathogenic | Pathogenic |
| c.853C > T | Nonsense | PVS1, PM2, PP3, PP4 | Pathogenic | Pathogenic |
| c.(871+1_872-1)_(*1_?)del | Large deletion | PVS1, PS3, PM2, PP4 | Pathogenic | Pathogenic |
| c.875insT | Frameshift | PVS1, PM2, PP3, PP4 | Pathogenic | Pathogenic |
| c.887C>A | Nonsense | PVS1, PM2, PP3, PP4 | Pathogenic | Pathogenic |
| c.890_893delAAAG | Frameshift | PVS1, PM2, PP3, PP4 | Pathogenic | Pathogenic |
| c.897G>A | Nonsense | PVS1, PM2, PP3, PP4 | Pathogenic | Pathogenic |
| c.906dupT | Frameshift | PVS1, PM2, PP3, PP4 | Pathogenic | Pathogenic |
| c.912delT | Frameshift | PVS1, PM2, PP3, PP4 | Pathogenic | Pathogenic |
| c.927_954dup | Frameshift | PVS1, PM2, PP1, PP4 | Pathogenic | Pathogenic |
| c.929_930insTT | Frameshift | PVS1, PM2, PP3 | Pathogenic | Pathogenic |
| c.932_933delCT | Frameshift | PVS1, PS3, PM2, PP4 | Pathogenic | Pathogenic |
| c.933delT | Frameshift | PVS1, PM2, PP3, PP4 | Pathogenic | Pathogenic |
| c.943G>T | Nonsense | PVS1, PM2, PP3, PP4 | Pathogenic | Pathogenic |
| c.946_947delAG | Frameshift | PVS1, PM2, PP3, PP4 | Pathogenic | Pathogenic |
| c.958dup | Frameshift | PVS1, PM2, PP3, PP4 | Pathogenic | Pathogenic |
| c.980insC | Frameshift | PVS1, PM2, PP3 | Pathogenic | Pathogenic |
| c.995_998del | Frameshift | PVS1, PM2, PP3, PP4 | Pathogenic | Pathogenic |
| c.997_998dup | Frameshift | PVS1, PM2, PP3, PP4 | Pathogenic | Pathogenic |
| c.998C>G | Nonsense | PVS1, PM2, PP4 | Pathogenic | Pathogenic |
| c.1014del | Frameshift | PVS1, PM2, PP1, PP4 | Pathogenic | Pathogenic |
| c.1015C > T | Nonsense | PVS1, PM2, PP3 | Pathogenic | Pathogenic |
| c.1021del | Frameshift | PVS1, PM2, PP1, PP4 | Pathogenic | Pathogenic |
| c.1062G>C | Intronic/splice site | PSV1, PM2, PP3, PP4 | Pathogenic | Pathogenic |
| c.1062+1G>A | Intronic/splice site | PVS1, PM2, PP1, PP4 | Pathogenic | Pathogenic |
| c.1062+2T>G | Intronic/splice site | PVS1, PM2, PP3, PP4 | Pathogenic | Pathogenic |
| c.1063-10_1065del | Intronic/splice site | PVS1, PS3, PM2, PP4 | Pathogenic | Pathogenic |
| c.1063-2A>G | Intronic/splice site | PVS1, PS3, PM2, PP4 | Pathogenic | Pathogenic |
| c.1063-151_1300+413dup | Large duplication | PVS1, PM2, PP3, PP4 | Pathogenic | Pathogenic |
| c.(1062+1_1063-1)_(1176+1_1177-1)del | Large deletion | PVS1, PM2, PP3, PP4 | Pathogenic | Pathogenic |
| c.1076delC | Frameshift | PVS1, PS3, PM2, PP4 | Pathogenic | Pathogenic |
| c.1093_1113del | Missense/in-frame deletion | PM2, PM4, PP3, PP4 | Likely pathogenic | Pathogenic |
| c.1117C>T | Nonsense | PVS1, PM2, PP3, PP4 | Pathogenic | Pathogenic |
| c.1127G>A | Nonsense | PVS1, PM2, PP3, PP4 | Pathogenic | Pathogenic |
| c.1135A>T | Nonsense | PVS1, PM2, PP3, PP4 | Pathogenic | Pathogenic |
| c.1153 C>T | Nonsense | PVS1, PM2, PP3, PP4 | Pathogenic | Pathogenic |
| c.1156_1175del | Frameshift | PVS1, PS3, PM2, PP1, PP4 | Pathogenic | Pathogenic |
| c.1165G > T | Nonsense | PVS1, PM2, PP3, PP4 | Pathogenic | Pathogenic |
| c.1176+1dupG | Intronic/splice site | PVS1, PS3, PM2, PP4 | Pathogenic | Pathogenic |
| c.1177-5_1177-3del | Intronic/splice site | PS3, PM2, PP2,PP3, PP4, PP5 | Pathogenic | Pathogenic |
| c.1177-2A>G | Intronic/splice site | PVS1, PM2, PP4 | Pathogenic | Pathogenic |
| c.1177-2A>C | Intronic/splice site | PVS1, PM2, PP3 | Pathogenic | Pathogenic |
| c.1177-10_1177-8delTCC | Intronic/splice site | PM2, PM4, PP3, PP4, PP5 | Pathogenic | Pathogenic |
| c.(1176+1_1177-1)_(1300+1_1301-1)del | Large deletion | PVS1, PM2, PP3, PP4 | Pathogenic | Pathogenic |
| c.1183_1198del | Frameshift | PVS1, PS3, PM2, PP4 | Pathogenic | Pathogenic |
| c.1215C>G | Nonsense | PVS1, PM2, PP3, PP4 | Pathogenic | Pathogenic |
| c.1219delA | Frameshift | PVS1, PS3, PM2, PP4 | Pathogenic | Pathogenic |
| c.1227C>G | Nonsense | PVS1, PM2, PP3 | Pathogenic | Pathogenic |
| c.1228G>T | Nonsense | PVS1, PM2, PP3, PP4 | Pathogenic | Pathogenic |
| c.1252delC | Frameshift | PVS1, PS3, PM2, PP4 | Pathogenic | Pathogenic |
| c.1273C>T | Nonsense | PVS1, PM2, PP3, PP4 | Pathogenic | Pathogenic |
| c.1283insG | Frameshift | PVS1, PS1, PS3, PM1, PM2, PP3 | Pathogenic | Pathogenic |
| c.1285C>T | Missense/in-frame deletion | PM1, PM2, PM6, PP1, PP4 | Likely pathogenic | Likely pathogenic |
| c.1285del | Frameshift | PVS1, PS1, PS3, PM1, PM2 | Pathogenic | Pathogenic |
| c.1285dupC | Frameshift | PVS1, PS1, PS3, PM1, PM2 | Pathogenic | Pathogenic |
| c.1286dup | Frameshift | PVS1, PS3, PM2, PP4 | Pathogenic | Pathogenic |
| c.1300G>A | Intronic/splice site | PVS1, PM2, PP4 | Pathogenic | Pathogenic |
| c.1300G>C | Intronic/splice site | PVS1, PS3, PM2, PP4 | Pathogenic | Pathogenic |
| c.1300G>T | Nonsense | PVS1, PM2, PP3, PP4 | Pathogenic | Pathogenic |
| c.1300+1G>A | Intronic/splice site | PVS1, PM2, PP4 | Pathogenic | Pathogenic |
| c.1300+2T>C | Intronic/splice site | PVS1, PM2, PP4 | Pathogenic | Pathogenic |
| c.1301-2A>C | Intronic/splice site | PVS1, PM2, PP3 | Pathogenic | Pathogenic |
| c.1301_1302delAG | Frameshift | PVS1, PS3, PM2, PP4 | Pathogenic | Pathogenic |
| c.1301-7_1304del; c.1323delinsGA | Frameshift | PVS1, PS3, PM2, PP4 | Pathogenic | Pathogenic |
| c.(1300+1_1301-1)_(*1_?)del | Large deletion | PVS1, PM2, PP3, PP4 | Pathogenic | Pathogenic |
| c.1305del | Frameshift | PVS1, PM2, PP1, PP4 | Pathogenic | Pathogenic |
| c.1318del | Frameshift | PVS1, PM2, PP4 | Pathogenic | Pathogenic |
| c.1318_1334dup | Frameshift | PVS1, PM2, PP4 | Pathogenic | Pathogenic |
| c.1337_1343dup | Frameshift | PVS1, PM2, PP4 | Pathogenic | Pathogenic |
| c.1340_1346dup | Frameshift | PVS1, PM2, PP3, PP4 | Pathogenic | Pathogenic |
| c.1347_1353dup | Frameshift | PVS1, PS3, PM2, PP4 | Pathogenic | Pathogenic |
| c.1367_1398del | Frameshift | PVS1, PM2, PP4 | Pathogenic | Pathogenic |
| c.1372dup | Frameshift | PVS1, PM2, PP4 | Pathogenic | Pathogenic |
| c.1379_1380delTC | Frameshift | PVS1, PM2, PP1, PP4 | Pathogenic | Pathogenic |
| c.1389C>G | Nonsense | PVS1, PM2, PP1, PP4 | Pathogenic | Pathogenic |
| c.1408delC | Frameshift | PVS1, PM2, PP3 | Pathogenic | Pathogenic |
| c.1408_1418del | Frameshift | PVS1, PM2, PP1, PP4 | Pathogenic | Pathogenic |
| c.1426dup | Frameshift | PVS1, PM2, PP1, PP4 | Pathogenic | Pathogenic |
| c.1429C>T | Nonsense | PVS1, PM2, PP1, PP4 | Pathogenic | Pathogenic |
| c.1432+1G>A | Intronic/splice site | PVS1, PM2, PP4 | Pathogenic | Pathogenic |
| c.1432+1G>T | Intronic/splice site | PVS1, PM2, PP4 | Pathogenic | Pathogenic |
| c.1433-1G>T | Intronic/splice site | PVS1, PS3, PM2, PP3 | Pathogenic | Pathogenic |
| c.1458del | Frameshift | PVS1, PM2, PP3 | Pathogenic | Pathogenic |
| c.1487_1490dup | Frameshift | PVS1, PM2, PP1, PP4 | Pathogenic | Pathogenic |
| c.1489_1490delGT | Frameshift | PVS1, PM2, PP3, PP4 | Pathogenic | Pathogenic |
| c.1522_1524delAAG | Missense/in-frame deletion | PM2, PM4, PM5, PM6, PP3, PP4 | Likely pathogenic | Pathogenic |
| c.1523A>G | Missense/in-frame deletion | PM6, PP1, PP3, PP4, PP5 | Likely pathogenic | Pathogenic |
| c.1528_1530delGAG | Missense/in-frame deletion | PM2, PM4, PP3, PP4, PP5 | Likely pathogenic | Pathogenic |
| c.1533G>A | Nonsense | PVS1, PM2, PP3, PP4 | Pathogenic | Pathogenic |
| c.1533_1536del | Frameshift | PVS1, PS3, PM2, PP4 | Pathogenic | Pathogenic |
| c.(1538+1_1539-1)_(*1_?)del | Frameshift | PVS1, PS3, PM2, PP4 | Pathogenic | Pathogenic |
| c.1539-2A>G | Intronic/splice site | PVS1, PM2, PP4 | Pathogenic | Pathogenic |
| c.1552delC | Frameshift | PVS1, PM2, PP3, PP4 | Pathogenic | Pathogenic |
| c.1564_1579delins | Frameshift | PVS1, PM2, PP3, PP5 | Pathogenic | Pathogenic |
| c.1557del | Frameshift | PVS1, PM2, PP3, PP4 | Pathogenic | Pathogenic |
| c.1579C>T | Nonsense | PVS1, PM2, PP1, PP4 | Pathogenic | Pathogenic |
| c.1579_1580insA | Frameshift | PVS1, PM2, PP3 | Pathogenic | Pathogenic |
| c.1596-1599delCA | Nonsense | PVS1, PM2, PP3, PP4 | Pathogenic | Pathogenic |
| c.1597C>T | Nonsense | PVS1, PS3, PM2, PP3 | Pathogenic | Pathogenic |
| c.1597_1598del | Frameshift | PVS1, PM2, PP3, PP4 | Pathogenic | Pathogenic |
| c.1653insTG | Frameshift | PVS1, PM2, PP3 | Pathogenic | Pathogenic |
| c.1658G > A | Nonsense | PVS1, PM2, PP3, PP4 | Pathogenic | Pathogenic |
| Deletion including exon 14 | Large deletion | PVS1, PS3, PM1, PM2, PP4 | Pathogenic | Pathogenic |

**Pathogenicity according to ACMG guidance** (1). PVS = Very strong, PS = Strong, PM = moderate, PP = Supporting

**ACMG class** – ‘likely pathogenic’ or ‘pathogenic’ classification according to ACMG guidelines (1)

**LOVD3 (Leiden Open Variation Database v.3.0)** – classification according to LOVD3 database of *FLCN* mutations (2).

**Supplementary References:**

1. Richards S, Aziz N, Bale S, Bick D, Das S, Gastier-Foster J, et al. Standards and guidelines for the interpretation of sequence variants: a joint consensus recommendation of the American College of Medical Genetics and Genomics and the Association for Molecular Pathology. Genet Med. 2015;17(5):405-24.

2. LOVD3 - Leiden Open Variation Database [Internet]. 2020. Available from: http://www.LOVD.nl/FLCN.
